# Spontaneous and Procedural Plaque Embolisation in Native Coronary Arteries: Pathophysiology, Diagnosis, and Prevention

**DOI:** 10.1155/2013/364247

**Published:** 2013-12-19

**Authors:** Giovanni Luigi De Maria, Niket Patel, George Kassimis, Adrian P. Banning

**Affiliations:** ^1^Oxford Heart Centre, John Radcliffe Hospital, Oxford University Hospitals, Headley Way, Oxford OX39DU, UK; ^2^Cardiovascular Medicine Department, Catholic University of the Sacred Heart, 00168 Rome, Italy

## Abstract

The detachment of atherothrombotic material from the atherosclerotic coronary plaque and downstream embolisation is an underrecognized phenomenon and it causes different degrees of impairment of the coronary microcirculation. During treatment of obstructive atherosclerotic plaque by percutaneous coronary intervention (PCI) distal embolisation (DE) is considered to be inevitable and it is associated with potential clinical and prognostic implications. This review aims to assess the main aspects of both spontaneous and procedural DE, analyze their different pathophysiology, provide specific insights on the main diagnostic tools for their identification, and finally focus on the main strategies for their treatment and prevention.

## 1. Introduction

Atherogenesis is the cause of ischemic heart disease and knowledge about its pathophysiology is progressively increasing. Progression of plaque within a vessel is not merely a consequence of cholesterol accumulation but a dynamic process related to a complex interaction of several risk factors [1]. Intermittent plaque erosion and healing are probably a common event but clinically occult unless protuberant thrombus either dislodges causing flow disturbance downstream or plaque volume increases during healing reducing the luminal volume. This reduced luminal volume may be too small and produces myocardial ischemia in the dependant territory during exertion, manifesting clinically as anginal chest pain.

Treatment of obstructive atheroma by coronary balloon angioplasty was originally described by Gruentzig in 1977. Since then progressive and remarkable innovations in techniques and materials have made percutaneous coronary intervention (PCI) a cornerstone for the treatment of obstructive coronary artery disease (CAD) in many clinical settings [2].

**Figure 1:**
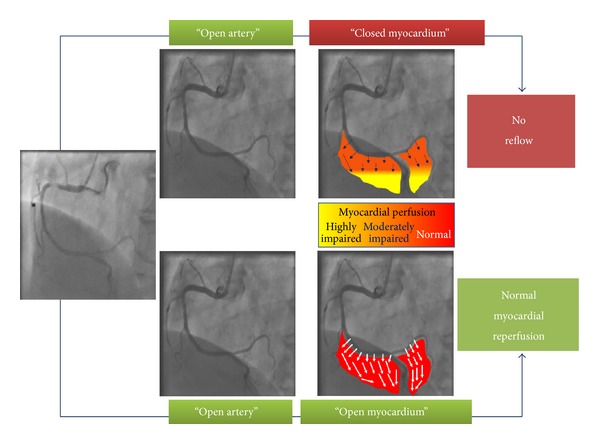
No reflow versus normal myocardial perfusion after revascularization.

However, achievement of epicardial coronary artery patency does not always translate into a complete and effective myocardial perfusion [3]. This phenomenon known as no reflow (NR) is caused by functional and mechanical impairment of coronary microcirculation and can be described as a condition of “open artery with closed myocardium” (Figure 1).

Individual susceptibility, ischemic-reperfusion, injury and distal embolisation (DE) are the main mechanisms leading to NR [4]. Among these three factors, DE of atherothrombotic debris downstream the coronary circulation with consequent microvascular obstruction (MVO) is the most relevant to the procedure of PCI. Its occurrence may prelude to a failure in restoring the normal myocardial blood flow with relevant clinical and prognostic implications [5, 6]. That is why understanding and preventing DE remain important for all clinicians and not just for interventional cardiologists.

This review article aims to shed light on this complex phenomenon, providing insights about the pathophysiology of DE and the available methods to detect and prevent it.

## 2. Distal Embolisation Definition and Pathophysiology

The term “distal embolisation” refers to the detachment of athero or atherothrombotic fragments from the atherosclerotic plaque and their dislodgement downstream the peripheral portion of the coronary tree. This leads to the occlusion of coronary microcirculation with consequent ongoing myocardial ischemia and necrosis. Compared to classical occlusion of an epicardial segment, DE produces patchy microinfarcts in the area at risk [7], with different degrees of myocardial injury [8].

DE was described for the first time in human in the 1980s by Falk and Davies in two series of patients with sudden death due to coronary thrombosis [9, 10]. The two groups reported that most of the thrombi had a layered structure, with thrombus material of different age. Intermittent thrombus fragmentation, with peripheral embolisation causing microembolic occlusion of small intramyocardial arteries associated with microinfarcts, was described in 73% of the cases [9, 10].

**Figure 2:**
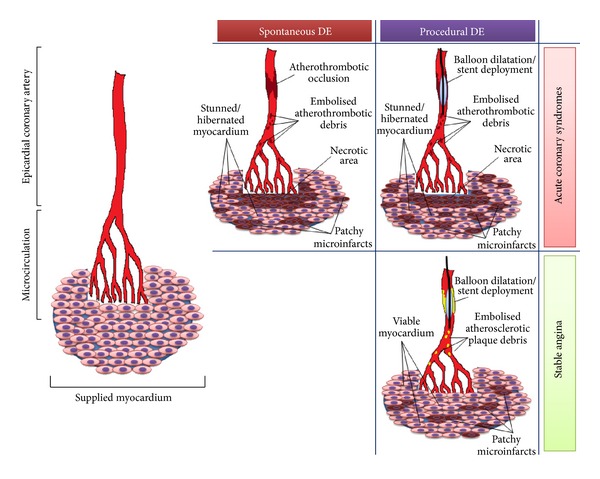
Mechanisms and consequences of “spontaneous” and “procedural” distal embolisation in stable coronary artery disease and in acute coronary syndrome. In spontaneous distal embolisation, occurring only in ACS patients, dislodgement of atherothrombotic debris contributes to enlargement of the area of myocardial injury and microvascular obstruction consequent to epicardial coronary occlusion. This damage is further increased by debris shower during revascularization procedure (procedural distal embolisation). In stable coronary artery disease only procedural distal embolisation occurs, causing patchy microinfarcts.

The interest in DE has been renewed after the development of PCI in which DE is related to balloon dilation or stent deployment. This allows labelling DE as “spontaneous” and “procedural” (Figure 2). Main differences between spontaneous and procedural DE in terms of pathophysiology and diagnostic and therapeutic tools are summarized in Table 1.

### 2.1. Spontaneous Distal Embolisation

Spontaneous DE is a phenomenon typically observed in ACS patients. It is strictly related to the pathophysiology underlying plaque instability, which is a progressive process going on for several days before the abrupt occlusion of the epicardial coronary artery. Late stage thrombi (>7 days), indeed, have been found in 69% of culprit plaques [11]. In this evolving phenomenon in which thrombosis and endogenous fibrinolysis take place simultaneously, thrombotic fragments from the growing up thrombus can be spontaneously dislodged and pushed downstream [8].

Broadly, spontaneous DE can be considered to be universal in coronary artery disease (CAD) and is a major component in processes accounting for ACS pathophysiology [7].

In terms of embolisate dimension, both macro- and microembolisation contribute to spontaneous DE. Macroembolisation refers to dislodgement of fragments with a diameter greater than 100 *μ*m, a cut-off derived from distal filter studies [8], and it consists of atherosclerotic fragments, fibrous and hyaline material, and mainly thrombotic debris [12].

Microembolisation refers to embolised debris with a diameter below 100 *μ*m. Platelet-platelets aggregates, neutrophils-platelets aggregates, monocytes-platelets aggregates [13], microthrombi [14], amorphous material [15], microparticles [16], cholesterol crystals [17], and leukocytes contribute to microemboli composition [18]. Most of the embolised material is classified as microembolisation [19]. Its consequences extend beyond its merely mechanical occluding effect as the embolic material has thrombogenic, vasoconstrictor, and inflammatory activity [15]. This pharmacological effect is also related to the occurrence of a humoral embolisation, since atherosclerotic plaques, especially if unstable and even ruptured or eroded, are rich in soluble factors able to cause or to worsen the microvascular damage during DE [20–23].

### 2.2. Procedural Distal Embolisation

Procedural DE is strictly related to PCI procedure and it can occur both in stable and ACS patients. It contributes to the pathophysiologic background of postprocedural myocardial infarction (Type 4a) [24].

Procedural DE recognises risk factors including hypertension [25], diabetes mellitus [26], preexisting renal failure, underlying ACS, lesion length and complexity, thrombus burden, interventions on saphenous vein graft (SVG), direct stenting versus predilation, number and duration of inflations, and rotablation or use of atherectomy [7]. Broadly an average 25% incidence has been described [7]; however, its occurrence is higher if microembolisation is taken into account beside the angiographically detectable macroembolisation.

Procedural DE composition varies from ACS to stable angina. In the ACS setting, procedural DE components are similar to those of spontaneous DE, mainly consisting of atherothrombotic debris, amorphous hyaline material (macroembolisation), foam cells, cholesterol crystals, platelet-platelet aggregates, leukocytes-platelet aggregates, microparticles, coagulation material (microembolisation) with a high content of tumor necrosis factor alpha, serotonin, thromboxane, and tissue factor (humoral embolisation) [15]. As for spontaneous DE, studies on filters adopted to prevent DE in primary PCI showed that most of procedural DE occurring in the unstable setting consists of microembolisation (22% particles <80 *μ*m, 30% 80–120 *μ*m, 16% 120–250 *μ*m, 15% 250–50 *μ*m, and 17% >500 *μ*m) [19].

In stable patients procedural DE consists mainly of fibrotic and hyaline material with few cells (foam cells and with a lesser degree also smooth muscular cells, neutrophils, and lymphocytes) [27]. Consequently the biochemical activity of procedural DE is less potent in stable CAD compared to ACS.

Clear evidence of procedural DE comes from intracoronary Doppler-flow-wire studies which reported firstly a typical reversal systolic flow with a delayed diastolic component and secondly a direct identification of macroemboli appearing as high intensity transient signals on the Doppler spectrum [28]. Further evidence for procedural DE comes from intravascular imaging, describing a direct relationship between plaque volume reduction after PCI with reduced myocardial reperfusion [29] and occurrence of postprocedural myocardial infarction and MVO in both stable and ACS patients [30, 31].

### 2.3. Distal Embolisation Pathophysiology

The relationship between myocardium and DE has been assessed in animal models with injection of inert 40 *μ*m microspheres. This infusion resulted in left ventricular impairment with inotropic reserve reduction associated with a compensatory increase of basal coronary blood flow [32] due to adenosine release from ischemic myocytes at the interface between embolised and non embolised myocardium [33]. The increased basal coronary flow caused a blunted vasodilation response, due to occluded small vessels after DE, and led to a significant reduction in coronary flow reserve at the site of embolised myocardium. This is confirmed in human by cardiac magnetic resonance (CMR) analysis [34].

This “contraction-perfusion” mismatch represents a peculiarity of DE.

**Figure 3:**
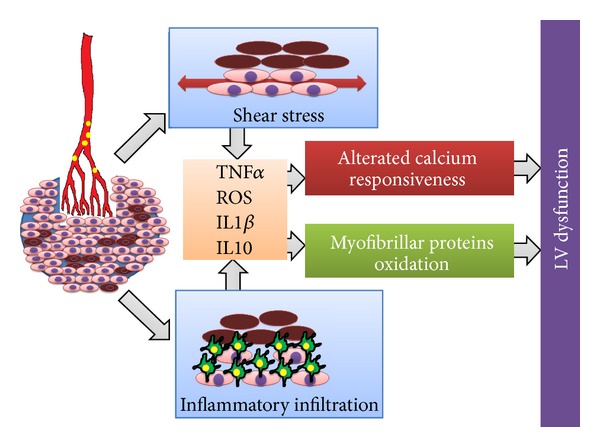
Mechanisms leading to left ventricular impairment after distal embolisation. Inflammatory response and shear stress at the interface between embolised and non embolised myocardium trigger the cytokines cascade which leads to myofibrillar proteins oxidation and reduced calcium responsiveness, underlying left ventricular dysfunction after distal embolization.

Both autopsy studies and CMR studies [35, 36] confirm that DE produces patchy microinfarcts responsible for a disproportionate impairment of left ventricular function beyond the actual amount of damaged myocardium [37] which can be quite small after DE (up to 5% of the perfusion territory) [38]. Surprisingly left ventricular impairment is related to the total area of nonperfused myocardium rather than to the volume of necrosis itself [39]. This phenomenon is due to the different mechanism of left ventricular impairment in case of DE, that is, the inflammatory response and tumor necrosis factor release at the interface between embolised and non embolised myocardium [40–43] (Figure 3).

It is likely that procedural DE interaction with microvasculature and myocardium is related to the clinical setting. Indeed in stable patients procedural DE, acting on a viable and “healthy” myocardium, causes an injury pattern close to that observed in animal models, thus accounting for the damage to a small myocardial volume (up to 5%). In ACS patients, procedural DE amplifies myocardial injury, acting on a severely ischemic and necrotic area which, after epicardial coronary artery occlusion, has suffered from the phenomenon of ischemia-reperfusion injury and previous spontaneous DE [36].

## 3. Distal Embolisation: Diagnostic Tools

Isolated spontaneous DE has been clearly described in postmortem studies on patients who died from sudden death due to coronary thrombosis [9, 10]. Differentiating between spontaneous and procedural DE in ACS patients is difficult, as these two events occur together in patients undergoing therapeutic PCI with associated procedural DE.

A number of technologies are available to indirectly assess the occurrence of DE, by detecting periprocedural myocardial injury and MVO (Table 1).

### 3.1. Biomarkers of Myocardial Damage

Troponins are proteins involved in the regulation of cardiac and skeletal muscle contraction, acting by modulating the interaction between actin and myosin. There are three cardiac troponin isoforms, namely, troponin C (calcium binding), I (inhibitory), and T (tropomyosin binding), and each is involved in a specific step of calcium mediated actin-myosin interaction [44].

Creatine kinase (CK) is an enzyme involved in the regulation of cellular metabolism, catalysing the conversion of creatine to phosphocreatine thus modulating adenosine triphosphate (ATP) intracellular levels. There are three different isoforms, whereas CK MB is the one expressed by myocardium [45].

Increased levels of troponin I or T and of CK MB isoform represent an easy way to detect myocardial injury. Evidence of troponin increase on admission in ACS patients is probably related to spontaneous DE. This is particularly relevant in late presenting patients with older intracoronary thrombi, intermittent epicardial artery occlusion, and showers of microemboli distally to the coronary microcirculation.

There is some debate about the relevance of elevation of biochemical markers of myocardial injury after PCI. Troponin is excellent for risk stratification in ACS and has an advantageous release profile. However, it may be oversensitive for assessing postprocedural myocardial injury, especially since the advent of high-sensitivity troponin assays [46]. At higher levels of troponin elevation a direct relationship between myocardial injury markers and occurrence of PCI-related myocardial damage has been demonstrated [47] and associated with a poorer prognosis [48]. Conversely, new areas of myocardial necrosis cannot be detected after very small troponin elevations after PCI [49].

CK MB elevation has a less complex relationship between myocardial injury and myocardial necrosis after PCI [49]. Indeed, in this regard, in the Cornell Angioplasty Registry a postprocedural troponin elevation in the absence of CK-MB increase was not related to increased in-hospital mortality [50]. Furthermore, the MICASA study showed that a better stratification between myocardial injury and infarction was possible adopting CK-MB and that, paradoxically, all patients undergoing complex PCI would have had a Type 4a MI diagnosis, according to the postprocedural troponin increase threshold (>3 times the 99th percentile of the upper reference limit) [51, 52]. Interestingly, to obtain the same degree of stratification as CK-MB, troponin increases thresholds for injury and infarction should be increased to >12 times and >40 times the 99th percentile of the upper reference limit, respectively [53]. Notably, these values are even beyond those reported by the recent Third Universal Definition of Myocardial Infarction (>5 times the 99th percentile of the upper reference limit) [24].

Moreover, it must be emphasised that postprocedural myocardial injury marker increase may not be entirely related to DE. Two patterns of postprocedural myocardial damage are described by CMR, namely, “distal” and “peristent.” Distal is directly related to procedural DE occurrence, whilst peristent reflects the occlusion by the stent of small branch vessels [31].

### 3.2. Contrast Echocardiography

Based on the infusion of small microbubbles able to remain completely within the microvasculature, myocardial contrast echocardiography (MCE) can detect areas of MVO as region of persistent contrast defect in the territory of the reopened culprit vessel [54]. Moreover, MCE would allow differentiating between procedural DE in stable CAD from the combination of both procedural and spontaneous DE occurred in ACS. Indeed, persistent contrast defect regions are typical of ACS patients in which a double embolic shower (spontaneous and procedural) occurs over an already severely compromised area of myocardium [54]. On the contrary, procedural DE in stable CAD patients does not produce contrast defects within the risk area probably because embolic debris does not lead to occlusion of all the arterioles [54].

High spatial and temporal resolution and the relatively low cost are the main advantages of MCE which is however an operator-depending technique [55]. It must be underlined that, at the moment, MCE is not indicated for clinical study but it remains a promising and useful research tool.

### 3.3. Cardiac Magnetic Resonance Imaging

Contrast-enhanced CMR represents, at the state of art, the gold standard for MVO assessment, with a good sensitivity, superior to MCE [56].

Two CMR strategies are available to identify MVO. In the first passage method, only after two minutes from contrast injection, MVO appears as hypoenhanced area while the whole myocardium results moderately hyperenhanced. This method is affected by low spatial resolution, reduced left ventricular coverage, and then low diagnostic sensitivity [56].

Higher spatial resolution, increased left ventricular coverage, and the least variability can be obtained with late gadolinium enhanced approach, in which image acquisition, after 10–15 minutes from contrast dye injection, allows detection of MVO as a hypoenhanced area within the hyperenhanced infarct region [57, 58].

Also the timing for CMR scanning has been shown to be relevant since only persistent MVO observed up to one month is really associated with worse regional wall motion and poor prognosis [59].

Notably CMR allows discriminating the mechanism of postprocedural myocardial injury. Indeed, two kinds of late gadolinium enhancement have been described after PCI. A so-called “persistent” pattern, in which the enhanced area is very close to the deployed stent, is caused by poststenting side branch occlusion. In comparison the “distal” pattern is described when the enhanced area is located at least 10 mm downstream from the stented siste and it is caused by procedural DE [31]. Higher poststenting plaque volume reduction and a significantly reduced myocardial perfusion reserve have been observed in areas of myocardium affected by post-stenting “distal” hyperenhancement [31, 34].

Despite cost, availability issues, and difficult performance in unwell patients, high specificity and sensitivity makes CMR the reference technique for the assessment of acute and chronic consequences of DE.

### 3.4. In the Cath-Lab

Typical electrocardiographic signs of DE have been identified in patients undergoing elective PCI and postprocedural myocardial injury defined as troponin increase [60]. While the surface ECG appears to be less predictive of DE, significant ST depression at intracoronary ECG recording presents a 74% sensitivity and a 95% specificity in detecting periprocedural troponin increase [60].

Moreover, evaluation of ST resolution at surface ECG has been easily applied to assess the occurrence of MVO and thus indirectly both spontaneous and procedural DE, in STEMI patients undergoing primary PCI [61].

TIMI flow grade [62] and corrected TIMI frame count (cTFC) [63] have been initially adopted as easy angiographic tools to assess postprocedural flow. However, neither TIMI flow grade nor cTFC can provide a direct assessment of tissue perfusion. Myocardial blush grade (MBG) is to date the main angiographic method to assess occurrence of microvascular impairment, related to DE [64]. MBG showed a significant relationship with ST resolution [61], left ventricular function recovery [64], arrhythmogenesis [65], and prognosis [66].

However, the main MBG limitation remains its interobserver variability which can be overcome with a new automated MBG quantification software (Quantitative Blush Evaluator (QuBE)) developed by Zwolle's group [67] and validated with CMR [68].

Intravascular imaging, namely, intravascular ultrasound (IVUS) and optical coherence tomography (OCT), by providing detailed insights into atherosclerotic plaque composition and structure, might be useful in the early identification of prone-to-DE lesions. In this regard, the presence of a necrotic core component derived from virtual histology-IVUS and the morphologic characteristics of plaque derived from grayscale IVUS have been shown to be closely related to procedural DE after PCI [69, 70].

Due to its recent introduction in the clinical arena, there is not enough evidence for OCT role in DE diagnosis. However, an additive predictive value of OCT for post-PCI CK-MB elevation in case of IVUS-echo attenuated plaques [71] has been reported. Moreover, a correlation exists between longitudinal plaque lipid pool assessed by OCT and MVO occurrence in STEMI patients [72].

The adoption of the pressure-thermistor-tipped guidewire might provide relevant details about the condition of coronary microcirculation. In this regard an increased index of microcirculatory resistance (IMR), defined as the ratio between distal pressure and the inverse of the hyperaemic mean transit time, has been recently related to poor long-term outcomes [73] showing a predictive value for MVO occurrence [74]. Similarly a worsening in IMR has been observed in elective patients who underwent PCI on prone-to-DE plaques at virtual histology [75].

Thus, even if their introduction in the clinical arena still needs confirmation from larger clinical trials, both intravascular imaging and coronary functional tests might find an application as diagnostic tools for DE detection and prevention.

## 4. Therapy of Distal Embolisation: Is Prevention Better Than Treatment?

**Figure 4:**
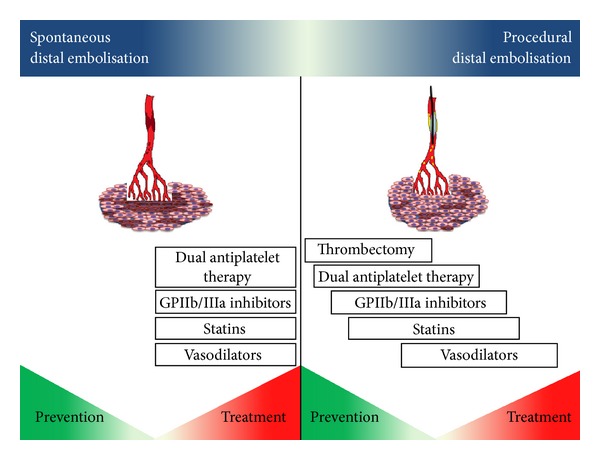
Prevention and treatment of spontaneous and procedural distal embolisation. Main mechanical and pharmacological options to treat and prevent distal embolisation in native vessels are reported according to their contribution in dealing with both spontaneous and procedural distal embolisation. Notably since spontaneous distal embolisation has already occurred or is ongoing at admission, pharmacological strategies are adopted with an only “therapeutic” intention in order to reduce the impact of embolised debris on myocardium. On the contrary the same drugs have a double role, preventive and therapeutic, in case of procedural distal embolisation.

Due to the relevant clinical impact of DE, especially in STEMI patients, several strategies, both pharmacological and mechanical, have been developed to prevent or to reduce the detrimental implications of DE. These strategies, mainly applied in ACS patients, are a useful tool to prevent procedural DE and to treat the consequences of spontaneous DE (Table 1 and Figure 4).

### 4.1. Spontaneous Distal Embolisation

Pharmacological strategies can be applied to prevent and mainly to limit the myocardial injury in case of spontaneous DE. However, the same pharmacological tools can be adopted to reduce the myocardial damage whenever prevention of procedural DE fails.

#### 4.1.1. Pharmacological Prevention

Three main categories of drugs are available: antiplatelet drugs, statins and vasodilators.

*(1) Antiplatelet Drugs*. By reducing the thrombotic burden all these agents can limit the occurrence and the amount of both spontaneous and procedural DE.

A direct effect of aspirin on prevention of DE has never been investigated before. However, recent data support a possible contribution of aspirin in DE prevention by reporting a higher thrombus burden [76] and a postprocedural lower coronary flow reserve [77] in ACS patients with documented aspirin resistance.

A direct benefit in terms of periprocedural myonecrosis reduction, and thus in terms of DE prevention, has been shown for P2Y12 receptors antagonists.

The reduction in thrombotic endpoints observed in the PCI-CURE [78], TRITON TIMI 38 [79], and PLATO [80] is the background to think about a role of P2Y12 receptors inhibitors, respectively, clopidogrel, prasugrel, and ticagrelor, in limiting both spontaneous and procedural DE occurrence.

The strength of antiplatelet activity appears to be dose- related for clopidogrel, as supported by the ARMYDA trials in which higher clopidogrel loading [81] and maintenance dose (600 mg and 150 mg, resp.) [82] were associated with a lower incidence of postprocedural myocardial injury. The more favourable pharmacokinetics and pharmacodynamics, instead, underlie the superiority of prasugrel and ticagrelor over clopidogrel observed in TRITON TIMI 38 and PLATO trials, in which a lower occurrence of myocardial infarction in the first days of treatment was likely associated also with an advantage in terms of reduced DE.

Similarly, the analysis of patient-level data from the three CHAMPION trials might support a role for cangrelor, a new fast-acting intravenous antiplatelet agent, in limiting the occurrence and the consequences of DE [83].

Besides P2Y12 receptors antagonists, also GPIIb/IIIa inhibitors may reduce downstream embolisation local generation of thrombus as well vasoactive and chemotactic mediators release from activated platelets [84].

A large core of evidence supports the application of GPIIb/IIIa inhibitors in high-risk PCI with evidence of highly thrombotic burden but a still open debate is about the best timing and the best route for their administration.

After the first evidence of major adverse cardiac events (MACE) reduction with preprocedural administration [85], a clear benefit from GPIIb/IIIa inhibitors upstream adoption has not been confirmed in larger trials [86, 87]. That is why, currently, guidelines recommend GPIIb/IIIa upstream use (versus in lab-use) only in high-risk patients transferred for primary PCI (IIb level B) [88].

The CICERO trial reported a better MBG and a lower postprocedural myonecrosis using intracoronary administration although no differences in the incidence of MACE were found [89]. The INFUSE AMI trial suggested benefit from intracoronary abciximab as an alternative or on top of manual thrombectomy, with a 11.8% reduction of infarct size assessed at CMR [90].

A local abciximab delivery strategy by adoption of specifically designed catheters has been purposed. The ongoing COCTAIL II trial will specifically address this issue [91], after that a small study on 50 patients has reported a greater thrombus burden reduction, a lower postprocedural cTFC, and a lower incidence of MACE at one year in patients who underwent local abciximab delivery compared to intracoronary infusion [92].

*(2) Statins*. Statins exert a stabilizing action on atherosclerotic plaque, protecting from coronary events and leading to a reduction of periprocedural myocardial damage occurrence [93]. Statins efficacy in reducing periprocedural myocardial injury is beyond their lipid-lowering action and is more likely related to the well-known pleiotropic effects [94, 95]. Notably, statins do not prevent DE itself, but they dramatically reduce the impact of embolised debris overt myocardium by attenuating inflammatory consequences of microembolisation. A lower index of myocardial resistance (IMR) in patients pretreated with statins [96] has also been reported.

*(3) Vasodilators*. Since DE causes both mechanical and functional microvascular impairment, vasodilators would act by dilating the obstructed microcirculation, counteracting the vasoconstrictive activity of embolisate. Two main vasodilators agents are currently used in the Cath-Lab to limit the impact of DE: adenosine and sodium nitroprusside.

Adenosine causes vasodilation by binding A2 receptors and inducing adenylate cyclase and platelets nitric oxide synthase activity. At the same time, adenosine exerts an anitinflammatory activity by hampering neutrophils accumulation, limiting free radicals production, damping endothelin-1 synthesis, and preventing intracellular Ca++ overload [8]. The first study to document a benefit from adenosine infusion was the AMISTAD I reporting an infarct size reduction in the adenosine group [97]. Even if the AMISTAD-II study broadly failed to detect a significant clinical advantage from intravenous adenosine, however, a benefit of adenosine infusion was reported in those patients treated earlier (within 3-4 hours) and with simultaneous infusion of GPIIB/IIIa [98].

Sodium nitroprusside is a potent endothelium independent vasodilator acting directly on vascular muscle cells, providing a longer maximal coronary hyperemia at a lower dose compared to adenosine [99]. In the only randomized study, nitroprusside showed a better clinical outcome even if not related to myocardial perfusion and coronary flow [100]. The recently published REOPEN AMI trial has shown a net benefit of intracoronary adenosine infusion after thrombectomy compared to nitroprusside and placebo in terms of ST resolution and angiographic detectable MVO [101].

The low nitroprusside dose adopted in the study and the large number of pleiotropic effects exhibited by adenosine are possible explanations for the superiority of intracoronary adenosine in terms of microvascular protection.

Other agents such as nondihydropyridine calcium channel blockers (verapamil and diltiazem), endothelin antagonists, and nicorandil have been purposed to rescue coronary microcirculation from DE, but their use in clinical daily practice is still limited due to lack of strong evidence.

Even if a clear advantage in terms of clinical outcomes has not been confirmed in larger clinical randomized trials, the benefit in terms of infarct size reduction and lower occurrence of MVO supports the current vasodilators adoption, especially adenosine, in case of spontaneous and procedural DE. As for GPIIb/IIIa, also for vasodilators, and again mainly for adenosine, there is a debate about the best time (before/during versus after PCI completion) and the best route of infusion (intracoronary versus intravenous) and ongoing studies will soon address this issue.

Broadly, most of the studies until now have mainly investigated adenosine use after procedure completion; however, since adenosine has a central role in myocardial conditioning [102], it is reasonable that its earlier and prolonged adoption could better prevent the deleterious effects of both spontaneous and procedural DE.

More debated is the issue about the best route of administration. Indeed after the first large clinical trials confirming the effectiveness of intravenous route, intracoronary adenosine administration has been shown to be safe and feasible [103], with a lesser degree of side effects thanks to a lower dosage compared to intravenous systemic infusion [104], and with a high cardioprotective activity [105] and improved tolerance to both spontaneous and procedural DE [106]. However, results from clinical randomized studies adopting intracoronary adenosine infusions are controversial [107, 108]. While a possible explanation could be the lack of agreement on the appropriate intracoronary adenosine dosage able to produce an effective myocardial hyperemia, further investigations are warranted to verify a real superiority of the intracoronary route.

### 4.2. Procedural Distal Embolisation

While pharmacologic approaches for prevention and treatment of procedural DE overlap with those of spontaneous DE, mechanical prevention is a strategy specifically addressed to prevent the occurrence of procedural DE.

#### 4.2.1. Mechanical Prevention

Balloon/filter protection devices and thrombectomy constitute the equipment for interventional cardiologist dealing with PCI in settings of higher risk of DE, namely, thrombotic lesions and degenerated SVG.

*(1) Balloon/Filter Protection Devices*. Balloon/filter devices consist of three main types: distal occlusion devices, distal filters, and proximal occlusion devices. Among them, distal filters, consisting of baskets placed distally to lesion, are the most used in clinical practice. Their adoption is however limited to PCI on SVG, since main clinical randomized trials failed to show a real clinical benefit in PCI on native vessels [2]. Embolisation during device deployment, a delay to PCI due to device deployment, and “filter no-reflow” phenomenon occurrence have been claimed as possible mechanisms explaining filters failure in PCI on native vessels [109, 110]. 

*(2) Thrombectomy*. Aspiration of thrombus material from the coronary lumen is the principle underlying thrombectomy. By providing thrombus debulking, thrombectomy significantly reduces the risk of procedural DE in highly thrombotic lesions and it could also account for a lower probability of spontaneous DE in case of STEMI patients treated with a “lone thrombus aspiration” approach (e.g., thrombus aspiration without stenting) [111].

This tool for DE prevention has currently a IIa indication according to European guidelines in STEMI patients [88]. After the negative result of the TATORT-NSTEMI trials [112], the ongoing TAPAS II [113] will clarify a potential role for thrombectomy also in NSTE-ACS with evidence of high thrombotic burden.

Two main kinds of thrombectomy devices are available at the moment: manual and mechanical. In manual thrombectomy devices, thrombus aspiration is obtained by application of a suction force exerted by the operator. In mechanical devices, aspirating force is produced by specific device related mechanisms.

Greater profiles, lower flexibility, and a steeper learning curve have been advocated as the main weak points of mechanical devices. Only two randomized trials have directly compared manual versus mechanical aspiration and both reported no differences between the two approaches in terms of MACE, highlighting however a higher rate of successful deployment of the manual thrombectomy device compared to the mechanical one [114, 115]. These data have also been independently confirmed in two meta-analyses [116, 117].

Recently, the interest for manual thrombectomy has progressively increased. After the first studies ascertaining its benefit in terms of myocardial reperfusion (assessed by ST resolution, MBG [118–125]), MCE [126, 127], and CMR [128] (Table 2), specific randomized clinical trials were needed to confirm its real clinical value.

Even if not designed to address this issue, the TAPAS was the first large study enrolling up to 1071 STEMI patients, reporting not only a better myocardial reperfusion in thrombectomy group [118], but also a reduced cardiac mortality and a lower MACE rate at one-year follow-up [129].

These results, however, have been recently debated in the TASTE trial which failed to find significant differences in terms of mortality, recurrent myocardial infarction, and stent thrombosis at thirty-day follow-up between manual thrombectomy group and controls [130]. The study has the merit to be the first, large (up to 7244 patients) trial designed to address the issue of clinical impact of manual thrombectomy. However, it must be underlined that the thirty-day follow-up could have been too short to assess a real benefit in terms of mortality and occurrence of left ventricular failure related to unfavourable remodeling. Moreover, final results might have been hampered by excluding high-risk patients (those who might have had a real benefit from thrombus aspiration). This apparent “draw” between TAPAS and TASTE studies might be solved after the conclusion of the large ongoing TOTAL trial (ClinicalTrials.gov number NCT01149044).

However, the TASTE trial could indirectly suggest that thrombectomy might really make the difference in specific settings featured by a higher risk of DE such as in patients with large thrombotic burden [131], extended myocardial area at risk [132], and prolonged time to treatment [133].

Finally the results from TASTE trial and TROFI study [134], which failed to detect a significant difference in terms of flow area and stent area between thrombectomy group and control group, might open the door to novel thrombus aspiration devices. ASPIRE aspiration systems, able to produce and to modulate higher aspiration forces, combined with larger-lumen catheters (MAX aspiration catheters), are actually under investigation and at the moment they have obtained FDA approval for application in peripheral vasculature [135].

## 5. Conclusions

DE needs to be considered in the pathophysiology of CAD and it represents a key event during PCI. Spontaneous and procedural DE can both lead to myocardial injury but the main clinical implications are in PCI and in STEMI patients. Several diagnostic tools are available to detect myocardial injury following DE and progress in intravascular imaging and functional intracoronary tests will be helpful in early detection of DE occurrence and upstream identification of those anatomical settings at higher risk of DE.

Thrombus aspiration, GPIIb/IIIa adoption, dual antiplatelet therapy, and intensive statin treatment are recommended to limit the impact of both spontaneous and procedural DE.

## Figures and Tables

**Table 1:** Main differences in pathophysiology, diagnosis, and prevention of spontaneous and procedural distal embolisation.

	Distal embolisation		
	Spontaneous	Procedural	
		Stable CAD	ACS
Clinical setting	ACS		

Embolisate dimension	Macro- and microembolisation	Macro- and microembolisation	

Embolisate composition	Atherothrombotic fragments Platelets aggregates Platelet-monocytes aggregates Microthrombi Microparticles Cholesterol crystal Amorphous material Humoral factors	Atherosclerotic fragments Hyaline material Fibrous material Cholesterol crystal	As in spontaneous DE

Biochemical activity	↑↑↑	↑	↑↑↑

Diagnosis	True diagnosis only at postmortem analysis	Laboratory ↑ Troponin ↑ CK MB Imaging MCE CMR Cath-Lab TIMI flow	Laboratory ↑ Troponin ↑ CK MB Imaging MCE CMR Cath-Lab Intracoronary ECG ST resolution TIMI flow and cTFC MBG

Therapy and prevention	Pharmacologic Aspirin Clopidogrel Prasugrel/ticagrelor GPIIb/IIIa inhibitors Statin Vasodilators (adenosine/nitroprusside)	Pharmacologic Aspirin Clopidogrel Statin GPIIb/IIIa* Vasodilators*	Pharmacologic As in spontaneous DE
	Mechanical none	Mechanical (only in SVG) Distal protection Balloon-based Filter-based Proximal protection	Mechanical thrombectomy

*Only in complicated PCI.

ACS: acute coronary syndrome; CAD: coronary artery disease; CMR: cardiac magnetic resonance; cTFC: corrected TIMI frame count; DE: distal embolisation; MCE: myocardial contrast echocardiography; MBG: myocardial blush grade; TF: tissue factor; TIMI: thrombolysis in myocardial infarction.

**Table 2:** Main results from clinical trials about manual thrombus aspiration compared to conventional PCI.

Study	Year	Device	PTS	Endpoint	FUP	Results	References
REMEDIA	2005	Diver CE	99	ST resolution ≥ 70% MBG ≥ 2 MCE index WMSI	X 6 months 6 months	↑ ↑ ↓ ↓	[101, 106]

DEAR MI	2006	Pronto	148	ST resolution ≥ 70% MBG ≥ 2	X	↑ ↑	[100]

De Luca et al.	2006	Diver CE	76	ST resolution ≥ 70% MBG ≥ 2 LV remodeling MACE	X 6 months 6 months	↑ ↑ ↓ *↔*	[102]

Chevalier et al.	2008	Export	249	ST resolution ≥ 70% MBG ≥ 2	X	↑ ↑	[105]

Chao et al.	2008	Export	74	Delta TIMI Delta MBG	X	↑ ↑	[103]

TAPAS	2008	Export	1071	MBG 0/1 Cardiac death reinfarction	X 1 year	↓ ↓	[98, 109]

Lipiecki et al.	2009	Export	44	Infarct size (SPECT) LV fraction (SPECT)	X	*↔* *↔*	[104]

Liictro et al.	2009	Export	111	ST resolution ≥ 70% MBG ≥ 2 MCE index WMSI	X 6 months	↑ ↑ ↓ ↓	[107]

EXPIRA	2009	Export	175	ST resolution ≥ 70% MBG ≥ 2 MVO at CMR	X 3 months	↑ ↑ ↓	[108]

PIHRATE	2010	Diver CE	196	ST resolution ≥ 70% MBG = 3	X	↑ ↑	[99]

TASTE	2013	Eliminate Expert Pronto	7244	All-cause mortality Recurrent MI Stent thrombosis	30 days	*↔* *↔* *↔*	[110]

CMR: cardiac magnetic resonance; FUP: follow-up; LV: left ventricular; MBG: myocardial blush grade; MCE: myocardial contrast echocardiography; MVO: microvascular obstruction; PTS: patients; SPECT: single photon emission computed tomography; WMSI: wall motion score index.

## Abbreviations

ACS: Acute coronary syndrome
CAD: Coronary artery disease
CMR: Cardiac magnetic resonance
DE: Distal embolisation
IMR: Index of microcirculatory resistance
IVUS: Intravascular ultrasound
MACE: Major cardiovascular event
MBG: Myocardial blush grade
MCE: Myocardial contrast echocardiography
MI: Myocardial infarction
MVO: Microvascular obstruction
NR: No reflow
NSTEMI: Non-ST elevation myocardial infarction
OCT: Optical coherence tomography
PCI: Percutaneous coronary intervention
STEMI: ST elevation myocardial infarction.
